# Changes and differences in school food standards (2010–2021) and free school meal provision during COVID‐19 across the UK: Potential implications for children's diets

**DOI:** 10.1111/nbu.12556

**Published:** 2022-05-12

**Authors:** Rebecca Louise McIntyre, Ashley J Adamson, Michael Nelson, Jayne Woodside, Shirley Beattie, Suzanne Spence

**Affiliations:** ^1^ Human Nutrition Research Centre, Population Health Sciences Institute Newcastle University Newcastle upon Tyne UK; ^2^ Fuse, the Centre for Translational Research in Public Health Newcastle University Newcastle upon Tyne UK; ^3^ Public Health Nutrition Research Ltd London UK; ^4^ Department of Nutrition and Dietetics King's College London London UK; ^5^ Centre for Public Health, Institute of Clinical Science A Queen's University Belfast Belfast UK; ^6^ Education Scotland Livingston UK

**Keywords:** COVID‐19, food–based standards, free school meals, nutrient‐based standards, school food standards, schools

## Abstract

This paper explores changes to school food standards from 2010, free school meal provision during the COVID‐19 pandemic across the UK and potential implications for children's diets. To obtain information on UK school food policies and free school meal provision methods we reviewed several sources including news articles, policy documents and journal articles. School food is an important part of the UK's health agenda and commitment to improving children's diets. Each UK nation has food‐based standards implemented, however, only Scotland and Wales also have nutrient‐based standards. School food standards in each nation have been updated in the last decade. Universal free school meals are available for children in the first 3 years of primary school in England and the first 5 years of primary school in Scotland, with plans announced for implementation of free school meals for all primary schoolchildren in Scotland and Wales. There is a lack of consistent monitoring of school food across the UK nations, and a lack of reporting compliance to the standards. Each nation differed in its response and management of free school meals during COVID‐related school closures. Further, there are issues surrounding the monitoring of the methods to provide free school meal support during school closures. The role of school food has been highlighted during COVID‐19, and with this, there have been calls for a review of free school meal eligibility criteria. The need for improved and consistent monitoring of school food across the UK remains, as does the need to evaluate the impact of school food on children's diets.

## INTRODUCTION

Childhood obesity is a major public health concern, with one third of UK schoolchildren aged 10–11 years reported to be overweight or obese in 2019/2020 (Health and Social Care Information Centre, 2020), and rates apparently increased further in 2020/2021, raising concerns about rising obesity rates following the COVID‐19 pandemic (BBC News, 2021b; NHS Digital, 2021). Overweight or obesity during childhood and adolescence may increase the risk of non‐communicable diseases in adulthood (Sanders et al., 2015). Further, a poor diet has been associated with overweight and obesity, with evidence suggesting dietary habits formed in childhood have the potential to continue into adulthood (Craigie et al., 2011; Simmonds et al., 2016).

The *Childhood Obesity: A Plan for Action* published in 2016 outlined plans to reduce childhood obesity in England within the next decade, including making school food healthier (HM Government, 2016). In 2018, *Childhood Obesity: A Plan for Action, Chapter 2* outlined plans to update school food standards, particularly to reduce sugar content (HM Government, 2018). In 2019, *Time to Solve Childhood Obesity* was published, highlighting issues surrounding childhood obesity. This report by the Chief Medical Officer contained recommendations to help achieve the government's goal to halve childhood obesity in England by 2030. These included the provision of healthy food options at appropriate prices in schools and implementing monitoring to ensure compliance to school food standards (Davies, 2019). The other devolved nations had similar policies and targets regarding childhood obesity (Department of Health, 2019; Scottish Government, 2018; Welsh Government, 2019b).

Evidence suggests school food policies may improve children's total diet and dietary behaviours in school, including reduced consumption of saturated fat and increased fruit and vegetable consumption (Micha et al., 2018; Spence et al., 2013). Following the removal of nutritional standards for school meals in the UK in 1980 (Education Act, 1980), an example of a key policy change to school food was the reintroduction of food‐ and nutrient‐based standards implemented by 2008/2009 in England (The Education [Nutritional Standards and Requirements for School Food] [England] Regulations, 2007). Adamson et al. (2013) discussed the reintroduction of food and nutrient‐based standards across the four UK nations from 2000 to 2013, when each nation had different school food policies (Adamson et al., 2013). This continues to be the case. There has been no detailed review from 2013 onwards. Since then, several key changes to school food have been implemented across the UK, which varied between each nation.

Across the UK, schoolchildren can receive free school meals (FSM) if eligible. Eligibility is means‐tested and enables children from more disadvantaged backgrounds to receive a free school lunch, although eligibility cut‐offs vary across the UK nations (Department for Education, 2018; Education Authority, 2020; Scottish Government, 2021b; Welsh Government, 2019a). In addition, in England, all children in the first 3 years of school are eligible for FSM regardless of family income, this is called universal infant FSM and in Scotland all children in the first 5 years of school receive universal FSM (Children and Families Act, 2014; Children and Young People [Scotland] Act, 2014; Scottish Government, 2021c), for the purpose of this paper, this will be referred to as universal free school meals (UFSM).

On 11 March, 2020 an outbreak of SARS‐CoV‐2, referred to as COVID‐19 was declared a pandemic by World Health Organization (2020). All UK schools closed from Friday 20 March 2020; however, vulnerable children and children of keyworkers (e.g. nurses, supermarket workers) were allowed to continue attending school (Long, 2020). Despite nationwide lockdown and school closures, each UK nation was required to continue FSM provision for all eligible children (Department for Education, 2020c; Department of Finance, 2020; Scottish Government, 2021b; Welsh Government, 2020b); each UK nation differed in their approach to provide FSM throughout the pandemic.

This paper explores changes to school food policies from 2010 and FSM provision during the COVID‐19 pandemic across the UK and the potential implications for children's diets. To obtain information on school food policies in the UK (implementation, monitoring and evaluation) and methods of FSM provision during the COVID‐19 pandemic we reviewed websites, news articles, government publications, policy documents and journal articles. Several key terms were used including “School food standards”, “COVID‐19” and “free school meals”, and searches covered the time period 2010 to 2022.

## SCHOOL FOOD STANDARDS AND UNIVERSAL FREE SCHOOL MEALS

This section provides an overview of each UK nation's school food standards and UFSM policies. Similarities and differences are summarised in Table 1 and examples of current standards are detailed in Tables 2 and 3.

**TABLE 1:** Current school food standards and universal free school meal provision: Comparison across the four UK nations (2010–2022)

	England	Scotland	Wales	Northern Ireland
Food‐Based Standards	Yes	Yes	Yes	Yes
Nutrient‐Based Standards	No	Yes	Yes	No
Universal Free School Meals	Yes	Yes (Plan to implement in all primary school children)	Yes (Plans announced to implement for all primary schoolchildren in next 3 years)	No

**TABLE 2:** Examples of currently implemented food‐based standards in the four UK nations (2010–2022)

Category	Specific food group	England (The Requirements for School Food Regulations, 2014)	Scotland (The Nutritional Requirements for Food and Drink in Schools [Scotland] Regulations, 2020)	Wales (Welsh Government, 2014)	Northern Ireland (2013) (Department of Education, 2013)
Starchy foods	Bread	Should be available everyday			
	Potato products (per week)	Starchy food cooked in fat or oil no more than twice each week	Chips, if served, must be served as part of a meal	Cooked in fat/oil must not be provided more than twice each week	Cooked in fat/oil must not be provided more than twice each week
Fruit and vegetables	Vegetables (per day)	One or more portions as an accompaniment	At least two portions	At least one portion (primary) two portions (secondary)	At least 2 portions of fruit and vegetables
	Fruit (per day)	One or more portions	At least one portion	At least one portion (incl. fruit juice)	
Meat, fish, eggs, beans and other non‐dairy sources of protein	Oily fish	At least once every three weeks	At least once every three weeks	At least twice every four weeks	At least once every four weeks
	Meat (per week)	A portion of meat or poultry on three or more days	No more than a total 175 g of specified meat (e.g. beef, pork) (primary)	Two or more days (primary)	Between two to three days (primary)
			No more than a total of 230 g of specified meat (secondary)	Three or more days (secondary)	Between three to four days (secondary)
Milk and dairy	Milk	Lower fat milk at least once a day lower fat	Milk must contain a total fat content less than 1.8 g per 100 ml	Plain milk – semi skimmed or skimmed.	Drinking milk must be available each day
Drinks	Fruit and vegetable juice	Maximum of 150 ml	Fruit juice not permitted	Only available at mealtimes (primary)	Unsweetened fruit and vegetable juice permitted
Foods high in fat, salt and sugar	Confectionary	None permitted			
	Deep‐fried food products (per week)	No more than two portions	No more than three times per week	No more than twice per week	No more than twice per week

**TABLE 3:** Current nutrient‐based standards (lunchtime, 2010–2022) across the UK nations by school type (primary and secondary)

Nutrient (minimum or maximum requirement)	England & Northern Ireland^a^	Scotland (The Nutritional Requirements for Food and Drink in Schools [Scotland] Regulations, 2020)	Wales (The Healthy Eating in Schools [Nutritional Standards and Requirements] [Wales] Regulations, 2013)
**Energy**	n/a	(Daily tolerance within 15%, weekly tolerance within 10%)	
(Primary)		518 kcal	530 kcal
(Secondary^b^)		745 kcal	646 kcal
**Total fat (Maximum)**			
(Primary)		20.1 g	20.6 g
(Secondary^b^)		29 g	25.1 g
**Saturated fat (Minimum)**			
(Primary)		6.3 g	6.5 g
(Secondary^b^)		9.1 g	7.9 g
**Total carbohydrate (Minimum)**			
(Primary)		69.1 g	70.6 g
(Secondary^b^)		99.3 g	86.1 g
**Free sugars/non milk extrinsic sugars (Wales) (Maximum)**			
(Primary)		10.4 g	15.5 g
(Secondary^b^)		14.9 g	18.9 g
**Fibre (Minimum)**			
(Primary)		6 g	4.2 g
(Secondary^b^)		9 g	5.2 g
**Protein (Minimum)**			
(Primary)		19.4 g	7.5 g
(Secondary^b^)		27.9 g	13.3 g
**Iron (Minimum)**			
(Primary)		3 mg	3 mg
(Secondary^b^)		4.4 mg	4.4 mg
**Calcium (Minimum)**			
(Primary)		165 mg	193 mg
(Secondary^b^)		300 mg	300 mg
**Vitamin A (Minimum)**			
(Primary)		150 μg	175 μg
(Secondary^b^)		187 μg	245 μg
**Vitamin C (Minimum)**			
(Primary)		9 mg	10.5 mg
(Secondary^b^)		11 mg	14 mg
**Folate (Minimum)**			
(Primary)		45 μg	53 μg
(Secondary^b^)		60 μg	70 μg
**Sodium (Maximum)**			
(Primary)		686 mg	499 mg
(Secondary^b^)		824 mg	714 mg
**Zinc (Minimum)**			
(Primary)		2.1 mg	2.5 mg
(Secondary^b^)		2.8 mg	2.8 mg

Neither England nor Northern Ireland currently have nutrient‐based standards in place.

Co‐educational secondary schools (Welsh standards are separated into boys, girls, and co‐educational secondary schools).

### England

There have been three major changes in England since 2010. Firstly, in 2013, a government commissioned report *The School Food Plan* was published emphasising the importance of a whole school approach and several recommendations. A whole school approach to food promotes a healthy food culture in schools, encouraging children to choose healthy foods and involves the entire school community (Food for Life, 2022). The recommendations outlined included: introduce food‐based standards only which dictates what types of food and drinks can be on offer in schools and how often they should be available, encourage school lunch uptake, all children in primary school should receive FSM and cooking should be incorporated into the curriculum (Dimbleby & Vincent, 2013).

Secondly, following this report, in 2014, *The Requirements for School Food Regulations* (The Requirements for School Food Regulations, 2014) removed the nutrient‐based standards, which were based on minimum and maximum levels of various nutrients that should be present in school food, due to cost and time in regards to implementation, monitoring compliance and limiting the creativity of school cooks (Dimbleby & Vincent, 2013). The ‘new’ standards aimed to build on previous standards and make them less complex and easier to implement in practice, be more specific and emphasise the importance of food variety available (Department for Education, 2014). Table 2 outlines examples of food‐based standards. Changes included: more specific requirements for fruit and vegetables (e.g. at least three different fruits and three different vegetables available each week) and at least one portion of starchy food available each week must be wholegrain (Department for Education, 2014). In 2016, the government committed to update the school food standards however, this remains an ongoing review (HM Government, 2016; HM Government, 2018).

Thirdly, a further key change was the implementation of UFSM for children in the first 3 years of primary school: reception, year 1 and year 2 (aged 4–7 years) (Children and Families Act, 2014). This is a universal, non‐means‐tested FSM entitlement for all children in these school years (Children and Families Act, 2014). UFSM aimed to provide all children with a free, hot, nutritious meal at lunchtime and in turn improve educational attainment and help families save money (Dimbleby & Vincent, 2013).

### Scotland

The updated standards for Scotland *The Nutritional Requirements for Food and Drink in Schools (Scotland) Regulations 2020* came into force from April 2021 replacing the previous 2008 standards (The Nutritional Requirements for Food and Drink in Schools [Scotland] Regulations, 2020). These include food‐ and nutrient‐based requirements. Key changes include: updated minimum and maximum levels of nutrients to be present in school meals, a daily tolerance for energy has been introduced (daily energy content must be within 15% of requirement of 518 kcal in primary and 745 kcal in secondary schoolchildren) alongside the existing weekly tolerance of 10%, different criteria apply in several food and drink standards and a weekly maximum limit for red and red processed meat now applies across the school (The Nutritional Requirements for Food and Drink in Schools [Scotland] Regulations, 2020). The ‘new’ standard for free sugars was reduced from the previous standards for non‐milk extrinsic sugars. Also, AOAC fibre is used in the 2020 standard which includes all carbohydrates that are not absorbed or digested by the body plus lignin (SACN, 2015), replacing non‐starch polysaccharides used in the previous standard, with an increase in the minimum requirement in primary and secondary schools (Scottish Government, 2008; The Nutritional Requirements for Food and Drink in Schools [Scotland] Regulations, 2020). Specific food‐ and nutrient‐based standards are detailed in Tables 2 and 3.

Similar to England, in 2015, the *Children and Young People (Scotland) Act, 2014* introduced UFSM for all children in the first 3 years of school: primaries one to three (aged 4–7 years) (Children and Young People [Scotland] Act, 2014). The aim is to ensure every child has the best possible start in life and encourage healthy eating habits from childhood (NHS Health Scotland, 2016). It was announced in March 2021 that all primary schoolchildren in Scotland will be eligible to receive free school breakfasts and lunches by August 2022 (Child Poverty Action Group, 2020; Scottish Government, 2021a). The Scottish Budget for 2022/2023 announced all children in the first five years of primary school now receive UFSM, however, UFSM provision for all primary schoolchildren has been delayed until ‘later in the parliamentary term’ (Scottish Government, 2021c).

### Wales

The most recent school food policy in Wales is *The Healthy Eating in Schools (Nutritional Standards and Requirements) (Wales) Regulations, 2013* (The Healthy Eating in Schools [Nutritional Standards and Requirements] [Wales] Regulations, 2013). These standards include both food‐ and nutrient‐based and have been developed from the Appetite for Life guidelines (Welsh Local Government Association, 2020a). Examples are included in Tables 2 and 3. Changes include: soft drinks and confectionery must not be available in schools, oily fish must be available at least twice in a 4 week period (previously no specific oily fish regulations) (The Education [Nutritional Standards for School Lunches] [Wales] Regulations, 2001; Welsh Government, 2014). In Wales, the nutrient‐based standards in secondary schools are split into single sex schools, and co‐educational schools (The Healthy Eating in Schools [Nutritional Standards and Requirements] [Wales] Regulations, 2013). Nutrient‐based standards are detailed in Table 3. Unlike England and Scotland, UFSM has not been implemented so FSM are provided on a means‐tested basis only (Welsh Government, 2019a). However, plans were announced in November 2021 for UFSM for all children in primary school to be implemented within the next 3 years (BBC News, 2021c).

### Northern Ireland

The *Food in Schools Policy 2013* is the most recent school food policy in Northern Ireland (Department of Education, 2013). This policy made it mandatory for schools to adopt a whole school approach to food and nutrition, and comply with the previous *Nutritional Standards for School Lunches and The Nutritional Standards for Other Food and Drinks in Schools* (Department of Education, 2013). These standards included: at least two portions of fruit and vegetables must be available each day, oily fish must be available at least once every 4 weeks and, cakes and biscuits must only be available as part of a meal at lunchtime (Public Health Agency, 2009). Examples of food‐based standards are included in Table 2.

In January 2020, a consultation was launched with a plan for changes to be introduced in September 2020, however, they have not yet been introduced and have been delayed due to the COVID‐19 pandemic. The key changes include at least three portions of fruit and vegetables to be available during lunch, and 50% of bread available as an accompaniment and 50% of sandwiches should be wholegrain (or high fibre). Also, only lower fat milk (semi‐skimmed or skimmed) should be available and processed red meat should only be available at most once per week in primary and twice per week in secondary schools (Department of Education, 2020a).

Northern Ireland does not have UFSM implemented, and FSM are only provided on a means‐tested basis (Education Authority, 2020). Each nation implements different FSM eligibility cut‐off points, and Northern Ireland, for example, has higher eligibility cut‐offs in comparison to England (Department for Education, 2018; Education Authority, 2020; Scottish Government, 2021b; Welsh Government, 2019a).

## MONITORING AND EVALUATION OF SCHOOL FOOD STANDARDS

This section considers the methods in place to monitor school food standards across the UK nations. Both England and Wales require school governing bodies to ensure school food standards are met (Davies, 2019; Department for Education, 2021b; Welsh Government, 2014). England has introduced the healthy schools rating schemes allowing schools to achieve a bronze, silver or gold award based on a number of factors including school food standards compliance, food education, physical education and promotion of active travel (Department for Education, 2019). In Wales, school governors are required to produce an annual report to indicate what action is being taken to promote healthy eating and drinking and indicate how this is being completed (Welsh Government, 2014). Also, both nations have introduced schemes to promote child health. Wales introduced a National Quality Award which is given to schools that meet specific criteria in areas related to a whole school approach which includes all food and drinks provided must meet school food regulations (Welsh Government, 2010). Wales also has Food in School Co‐ordinators that support and advise local authorities (LAs), schools and caterers on how to comply with regulations, oversee nutritional analysis of food served in schools and work alongside Welsh Network of Healthy School Schemes and Estyn (Welsh Local Government Association, 2020a). In addition, England and Wales both have inspection bodies that assess quality and standards in education and training called Ofsted (England) and Estyn (Wales) which assess how schools support children's understanding of healthy eating and fitness but do not monitor compliance to school food standards (Estyn, 2017; School Food Plan, 2020). In February 2022, *Levelling up the United Kingdom* was published and outlines plans to improve compliance to school food standards (HM Government, 2022). A joint project between Department for Education and Food Standards Agency was announced piloting new methods to support and improve compliance to school food standards across LAs in England. Schools will be encouraged to provide information on their whole school approach to food on their school website, with plans for this to eventually become mandatory. Further, school governors will be provided with training to support a whole school approach and food teachers will receive funding and support to ensure children can leave school knowing how to cook six recipes to support healthy eating (HM Government, 2022).

In Scotland, LAs are required to demonstrate compliance with food‐ and nutrient‐based standards, monitored by Health and Nutrition Inspectors. This is done by monitoring all ingredients and items served against the standards. Nutritional analysis software allows school meal providers to demonstrate compliance with nutrient‐based standards (Education Scotland, 2020). External evaluation is completed in a sample of schools each year through Education Scotland's school inspection programme. Unlike the other UK nations, Scotland has Health and Nutrition Inspectors with a specialist background in nutrition who undertake this role on inspection teams (Education Scotland, 2020).

In Northern Ireland, individual schools are encouraged to self‐monitor and have been provided a checklist to assist, but this is not monitored by the Department of Education. Education Authority school catering services are responsible for regularly reporting to the Department of Education on compliance with the Nutritional Standards (Department of Education, 2013; Public Health Agency, 2009).

Across the UK, there is no consistent assessment, monitoring or reporting of compliance with the standards for school food.

## COVID‐19 RESPONSE AND SCHOOL MEALS

Following school closures due to COVID‐19 in March 2020 the government sought ways to support children entitled to FSM. Similarities and differences between FSM provision during the COVID‐19 pandemic in each UK nation are summarised in Table 4.

**TABLE 4:** The response by government to provision of free school meals (FSM) across the four UK nations during the COVID‐19 pandemic and related school closures (2020–2021)

	England	Scotland	Wales	Northern Ireland
FSM Delivery Methods	Vouchers (national scheme)	Vouchers	Vouchers	Direct bank transfer
	Food parcel	Food parcel	Food Parcels	
	Alternate vouchers	Direct bank transfer	Direct bank transfer	
		Other alternative methods considered		
Payment/Voucher Amount Received	£15 per week	Varies dependent on local authority	£19.50 per week	£13.50 per week
Summer Holiday Provision Confirmed	16 June 2020 (The Guardian, 2020c)	16 June 2020 (Scottish Government, 2020b)	22 April 2020 (Welsh Government, 2020c)	19 June 2020 (BBC News, 2020b)
Summer Holiday Provision Method	Voucher	Vouchers	Voucher	Direct bank transfer
		Food Parcel	Food parcel	
		Direct bank transfer	Direct bank transfer	
Further Holiday Provision Confirmed	8 November 2020 (until Easter 2021) (Department for Work and Pensions, 2020)	20 October 2020 (Until Easter 2021) (Scottish Government, 2020c)	15 October 2020 (Until Easter 2021) (Welsh Government, 2020a)	22 October 2020 (For October holiday) (Department of Education, 2020b), 19 November (until Easter 2022) (Northern Ireland Executive, 2020)
Holiday Provision amount	£15 per week	Varies depending on local authority	£19.50 per week	£13.50 per week
Return to School Date (after initial 2020 UK‐wide lockdown)	1 June for reception, year 1 and year 6 15 June for year 10 and year 12 (Department for Education, 2020a)	12 August 2020	29 June 2020 (only a third of children at one time) (BBC News, 2020d)	24 August 2020 for primary 7, year 12 and year 14
	All students from September 2020 (Department for Education, 2020d)	Full‐time return from 17 August 2020 (The City of Edinburgh Council, 2020)	Full‐time return from 1 September 2020 (Welsh Local Government Association, 2020b)	Full‐time return from 31 August 2020 (Department of Education, 2020c)

### England

During school closures, schools continued to receive expected funding including for UFSM and means‐tested FSM (National Audit Office, 2020). Schools were expected to continue providing support for children eligible for means‐tested FSM, although there was no requirement for schools to continue to provide for children that received UFSM (though some schools provided FSM support for these children), unless the child still attended school despite closures. Parents of children who usually receive UFSM could apply to receive means‐tested FSM support during school closures (National Audit Office, 2020). Eligible children received support worth £15 per week through either a food parcel, a Department for Education National Voucher Scheme or alternate voucher for local shops or supermarkets (National Audit Office, 2020).

FSM support was continued through the Easter and May half‐term school holidays for eligible children (Long, 2020). Children eligible for means tested FSM received vouchers using the National Voucher Scheme worth £15 per child per week (Department for Education, 2020b). Initially, there was no plan to continue provision during the 2020 summer holidays, but following extensive campaigning by footballer Marcus Rashford, Sustain and Good Law Project, the government announced that support would continue over the summer (BBC News, 2020a; The Guardian, 2020c). A further campaign for FSM support during school holidays until Easter 2021 was rejected by MPs following a parliamentary debate on 21 October 2020 (The Guardian, 2020a). As a result, numerous businesses and charities committed to provide meals for FSM eligible children during the October school holidays (BBC News, 2020e). Additionally, councils throughout England pledged to provide FSM support during October half‐term including Birmingham City council, and Lewisham council (The Guardian, 2020a). The government agreed in the Winter Spending Review to the COVID Winter Grant Scheme which allowed LAs the ability to distribute support to the families who need it over the 2020 Christmas holidays, and provided support until Easter 2021 (Department for Work and Pensions, 2020).

During the initial opening of schools in June 2020 for specific year groups, school meals were only expected to be available to all children attending school and school kitchens were expected to be fully opened by September 2020 (Department for Education, 2020a, 2020d). The School Food Plan Alliance released a checklist to provide guidance for school catering, particularly for schools with inhouse catering and included guidance for headteachers and planning how food would be served. For example, use of packed lunches during initial reopening was encouraged, differing from the usual combination of hot and cold lunches and discouragement of children from bringing food from home (School Food Plan Alliance, 2020).

### Scotland

LAs were issued with guidance by government on how to safely provide FSM during school closures including the use of school facilities to act as food preparation/delivery hubs (Scottish Government, 2020a). The methods by which FSM were provided to eligible children differed between LAs. Some LAs provided payments into bank accounts of eligible families (e.g. City of Edinburgh council provided £22.50 per child fortnightly; The Scottish Sun, 2020), others implemented use of supermarket vouchers (e.g. in Glasgow and Aberdeen each eligible child received £20 and £25 respectively each fortnight; The Scottish Sun, 2020) or other alternatives including food parcels to meet local needs (Scottish Government, 2020a) (e.g. in Falkirk children received Grab n Go packed lunches; The Falkirk Herald, 2020). Children in the first three years of school only received FSM if eligible by means‐testing, children receiving UFSM did not receive this (Scottish Government, 2020a).

On return to school in August 2020, LAs put plans in place for providing school food. Lunchtimes were staggered to avoid large groups, secondary schoolchildren were instructed to maintain social distancing when possible and school meal menus were limited (The City of Edinburgh Council, 2020). Food provision on return to school varied dependent on LA: Edinburgh council planned for no hot lunch to be provided until kitchens fully re‐opened and packed lunches were provided for FSM children and on home learning days direct payments were used (The City of Edinburgh Council, 2020).

FSM provision continued over the 2020 Easter and Summer holidays for children in primary and secondary school eligible for means‐tested FSM (Scottish Government, 2020b). As with term‐time support, methods of provision differed between LAs. Methods (per child) included: packed lunches or hot meal picked up from school (e.g. in West Lothian; West Lothian Council, 2020), supermarket vouchers (e.g. Aberdeen offered £25 vouchers fortnightly; Aberdeen City Council, 2020) or payment into family bank accounts (e.g. Glasgow offered a £80 one‐off payment; [Glasgow Times, 2020]). On the 20 October 2020, the Scottish government announced that LAs would be provided with funding to continue means‐tested FSM provision for eligible children during school holidays until Easter 2021. LAs could decide on mode of provision (Scottish Government, 2020c).

### Wales

FSM provision differed between LAs (Welsh Government, 2020b). Methods included: vouchers for local supermarkets worth £19.50 per week for each eligible child, delivery of food parcels to eligible families containing ingredients and instructions or direct bank transfer to eligible families worth £19.50 per week (Welsh Government, 2020b). The method of delivery differed slightly between LAs, including weekly food boxes (e.g. Blaneau Gwent council; Blaenau Gwent County Borough Council, 2020), direct bank transfer (e.g. Blaneau Gwent council changed to this method; Blaenau Gwent County Borough Council, 2020) or vouchers (e.g. Vale of Glamorgan council offered £39 vouchers fortnightly; Wales Online, 2020).

Following return to school part‐time in June 2020 and full‐time in September 2020, FSM support was provided to eligible children, and schools introduced social distancing measures including staggered lunch times (BBC News, 2020d; Welsh Local Government Association, 2020b). LAs were given the authority to determine school meal arrangements dependent on when schools were returning and the method of re‐opening (BBC News, 2020d), for example, schools within Torfaen varied in respect to catering service available, some schools provided hot meals and others provided packed lunches only until canteens fully reopened (Torfaen County Borough, 2020).

FSM support was continued over the Easter holidays and Wales was the first UK nation to confirm school meal provision over summer holidays for all eligible children (Welsh Government, 2020c). The methods used to provide support over the holidays included supermarket vouchers worth the equivalent of £19.50 per week, food parcels or direct bank transfer for each eligible child (Welsh Government, 2020b). The Welsh Government announced on the 15th October 2020 that FSM provision during school holidays would continue until Easter 2021 (Welsh Government, 2020a).

### Northern Ireland

FSM support was provided to eligible families using direct payment of £27 fortnightly per each eligible child into bank accounts (Department of Finance, 2020). Following schools reopening in August 2020, schools were encouraged to consider if children should stay in their classroom to eat lunch, use staggered lunch times, use outdoor facilities and a reduced menu with a choice of hot meals or packed lunches (Department of Education, 2020c).

FSM support continued over the 2020 Easter holidays. Northern Ireland was the last UK nation to confirm alternate school meal provision for the summer holidays on 19 June. As with term‐time provision, families received £27 fortnightly per child, paid fortnightly by direct payment into family bank accounts (BBC News, 2020b, 2020c). The continuation of FSM provision during the October school holiday was announced on 22 October 2020 (Department of Education, 2020b). The Department for Education announced on 19 November that holid ay FSM provision would continue until Easter 2022 including Christmas 2020 (Northern Ireland Executive, 2020).

## DISCUSSION

School food remains an important part of the UK's public health agenda and commitment to improving children's diets and reducing childhood obesity (HM Government, 2016, 2018). All four nations have food‐based standards, however, only Scotland and Wales also have nutrient‐based standards. Each nation has updated their standards within the last 10 years: Scotland has implemented new standards this year and England and Northern Ireland are currently reviewing standards. UFSM has been implemented only for children in the first 3 years of school in England and Scotland, with plans for Scotland to introduce this for all primary schoolchildren by August 2022. Plans have been announced to introduce UFSM for all primary schoolchildren in Wales within the next 3 years. England and Wales have similar approaches for monitoring and evaluation of school food standards, while Scotland and Northern Ireland's approaches are different. There is a lack of consistent monitoring of school food standards across the UK, and a lack of reporting compliance. Scotland alone has maintained systematic school food monitoring which includes independent external monitoring by a specialist as part of school inspections.

Studies that have explored the impact of school food standards are primarily limited to England, but, to date, none have examined the impact of the updated standards nor compared the impact of the different nutrient‐based and food‐based‐standards on dietary intakes in the UK. In England, although studies have explored the impact of school food standards on children's diets, most studies explored the impact of the previous standards rather than those introduced in 2014. For example, one study looked at the impact of previous school food and nutrient standards on the diets of children in primary schools, and found improvements in children's diets, for example reduced intakes of fat and saturated fat (Spence et al., 2013). However, a study looking at secondary schools found there was limited evidence of a positive impact of school food standards on overall diet (Spence et al., 2014). A qualitative study carried out in Wales involving school staff and LA members found that although school food standards implemented in secondary schools contributed to children's health and dietary intake, following introduction of updated standards, school meal take‐up decreased and pupils sought out their preferred food items out of school (Addis, 2019). In Northern Ireland, policy changes were more successful in primary schoolchildren than post‐primary, pupils consumed more healthy foods and less foods high in fat, sugar, or salt at break times (and overall). However, children in secondary schools were more likely to buy less healthy food and drinks from school than children in primary school (Public Health Agency, 2016). Generally, without adequate and consistent monitoring and evaluation of school food standards, it is difficult to determine if school food complies with regulations and how school food policies impact on the diets of children in both short‐and long‐term.

As previously mentioned, England and Scotland currently offer UFSM only to children in the first 3 and 5 years of school respectively. This differs from the recommendation made for all children attending primary schools in England to receive UFSM (Dimbleby & Vincent, 2013). The Children's Food Campaign has campaigned for FSM for all children in primary school, emphasising the potential benefits including: ensuring all children have access to healthy meals and potentially improved educational attainment associated with healthier diets (Sustain, 2020). Generally, evaluations of UFSM in both England and Scotland have found increased school meal uptake in the targeted year groups. An evaluation of UFSM in England found that prior to introduction of UFSM, take up of school meals was 38% and, one‐year post‐implementation, was 80%. Further research indicated numerous benefits of UFSM, including financial benefits for parents (average of £10 per week saved) (Sellen & Huda, 2018) and improvements in bodyweight outcomes for families not previously eligible for means‐tested FSM (Holford & Rabe, 2020). An evaluation of UFSM in Scotland was published in October 2016. There was no information on uptake of FSM specifically prior to implementation of UFSM. However, uptake of all school meals (free and paid) in primary schoolchildren increased post‐implementation of UFSM from 53.2% in 2014 to 64.6% in 2015. For UFSM specifically, uptake increased in the second year post‐implementation of UFSM from 78.9% in 2015 to 81.7% in 2016 (NHS Health Scotland, 2016). The increase in UFSM uptake, paired with potential for improvements in children's diets as a result of school food regulations would likely be beneficial, especially if UFSM is introduced for all children in primary school as currently planned in Scotland. Additionally, implementation of UFSM in primary schools in Wales and Northern Ireland could be beneficial to improve the diets of children, especially in school, and show similar benefits as found in a pilot evaluation of UFSM in England.

The Jamie Oliver Food Foundation published *A Report on the Food Education Learning Landscape* in 2017 and found wide variations in schools adopting a whole school approach to food. The report focused on England and noted that the school food environment is relatively unhealthy, particularly in secondary schools, with unhealthier food being available throughout the school day. The report also highlighted that monitoring of the school food standards was not as common in secondary as in primary schools and unhealthy snacks tended to be used as treats, rewards and for fundraising events contradicting the food education and healthy eating messages. The report outlined recommendations including: the government should make school food standards compulsory in all schools, development of continuing professional development courses specifically for delivery of food education and compulsory evaluation of food provision and food environment in schools (Jamie Oliver Food Foundation, 2017). Achieving these recommendations would likely be beneficial for improving the quality of school food, especially by improving monitoring to ensure compliance to standards.

A report by Guys and St Thomas' Charity *Serving up children's health* focused on schools in London, finding that 73% of schools visited had menus complying with school food standards. However, this did not always translate into meals that children chose to eat, with children choosing the more unhealthy options when available (Guys & St Thomas' Charity, 2020). The report also highlighted the need for formal monitoring of food served in schools throughout the school day to avoid inconsistency across schools (Guys & St Thomas' Charity, 2020). The *State of the Nation: Children's Food and Health* report published by Food for Life in 2019 found at least 60% of secondary schools may be non‐compliant with school food standards and also highlighted the importance of monitoring school food standard compliance (Food for Life, 2019).

Each UK nation differed in their response and management of FSM during COVID‐19 and related school closures. All schools closed at the same time, however, return to school and the method of returning differed by nation. Due to COVID‐19, schools and LAs were required to adapt their approach to school meals (particularly FSM) to ensure support for all eligible children. Each nation took a different approach to providing FSM to eligible children; England, parts of Scotland and Wales offered vouchers and food parcels; Northern Ireland and other parts of Scotland and Wales offered direct bank transfers. A rise in COVID‐19 cases meant that the UK once again entered lockdown and schools were ordered to close in January 2021. Previous methods of providing FSM dependent on local needs (e.g. food vouchers or parcels) were utilised during this period (BBC News, 2021a; Department for Education, 2021a; Department of Education, 2021).

There were major challenges in the monitoring of the methods to provide means‐tested FSM to eligible children during the COVID‐19 pandemic. Monitoring of supermarket vouchers is limited, with most monitoring focused on voucher uptake, and not purchases. Regarding vouchers, there is a lack of a feasible method to identify supermarket purchases; vouchers were to be used to buy food but relied on supermarket staff to ensure vouchers were used on food (National Audit Office, 2020). Also, as a result of initial delays in the National Voucher Scheme, some schools had to supply children with emergency food parcels to ensure they received support (BBC News, 2020f). There were media reports that many food parcels provided by schools were inadequate and did not meet school food standards with parents criticising a lack of fruits and vegetables and poor portion sizes (The Guardian, 2020b; The Sun, 2020). A 2017 study looked at reducing food insecurity using food vouchers or free daily lunch and found the daily lunch method to be preferable for many families, with less stigma associated with this method in comparison to food vouchers (Dalma et al., 2018). As with the supermarket vouchers, monitoring use of support for food provision provided through direct bank transfer is not possible due to payments being made directly into bank accounts.

There are limited studies exploring FSM provision during these school closures. One study reported approximately 51% of eligible children in the UK received FSM support (Parnham et al., 2020), suggesting government attempts to supply FSM to children in need during school closures was limited. Further, use of food banks has risen, with the Independent Food Aid Network reporting a 177% increase in provision of emergency food parcels in May 2020 compared to May 2019 (IFAN, 2020). It also reported an 85% increase in children supported by emergency food parcels (IFAN, 2020). The increased requirement of support could be due to several many factors, including delays in FSM support during school closures and reduced parental income due to furlough or loss of employment. This may have an ongoing impact on the diets of children (and their families) both throughout and post the pandemic.

There are few studies that explore the provision of school meals (both free and paid) following the reopening of schools. A study by Rose et al. (2021) looked at children aged 11–18 years views (plus the views of parents and school staff) on how COVID‐19 impacted school food provision following schools reopening. It was found that, in terms of food available, reduced menus items resulted in lack of choice, limited or no hot meals and participants thought that school food was less healthy compared with pre‐pandemic options. This included limited salad options, pizza served frequently, and limited fruit and vegetable options (Rose et al., 2021). More studies are required to explore the impact that COVID‐19 has had on school food and children's diets across the UK.

The National Food Strategy outlines recommendations particularly for disadvantaged children in England (Dimbleby, 2021). The strategy highlights FSM eligibility criteria should be expanded to allow more children from low‐income families to be included and so potentially improve children's diets. The strategy also recommends expanding the Holiday Activity and Food programme to include all of England to allow FSM eligible children to receive support during school holidays, following publication of *National Food Strategy: Part One* this was implemented nationwide for school holidays in 2021 (Dimbleby, 2021). These recommendations target key areas to provide adequate nutrition to children from more deprived households, particularly during the current global pandemic. This will be particularly important to families where parents have become unemployed or suffered reduced income.

## CONCLUSION

Although there are similarities in school food standards across the four nations, differences exist. In particular, Scotland and Wales have food‐ and nutrient‐based standards, England and Northern Ireland implemented food‐based standards only. Further, only England and Scotland currently offer UFSM to all children in the first 3 years of school (aged 4–7 years), with Scotland providing UFSM for all children in the first 3 years of school and plans in place to expand this into all children in primary school. However, plans have been announced to introduce UFSM for all primary schoolchildren in Wales. In terms of the COVID‐19 pandemic, all UK nations provided support for FSM eligible children, although methods and monetary value of provision varied. The pandemic has also highlighted pre‐existing issues including FSM eligibility criteria. There are several gaps in the evidence and areas for future research, for example, there is a lack of studies exploring the impact of school meals (both free and paid) on children's diet in UK, particularly in the devolved nations, and the impact of COVID‐19 on children's diets. Further, there is a lack of evidence for the consequences of the different standards across the four UK nations, these differing standards have the potential to cause confusion and have different impacts on children's diets across the UK. Comparative studies investigating which approach works best to increase intake of healthier foods, for example food parcels, vouchers or bank transfer, would also be helpful.

There are several potential implications for policy and practice. This review has explored changes to school food policy and methods used by UK nations to monitor provision. Lack of consistent monitoring by the government and/or LAs limits the ability to evaluate the potential impacts of school food policy and ensure compliance with school food standards. There is increased need for monitoring of school food within the context of other changes in the food system, for example, as food poverty issues continue, and related issues emerge around food procurement. Lessons learned from the COVID‐19 pandemic highlight the need for forward planning or policies to protect the diet of the most vulnerable children in the event of a pandemic and subsequent school closures.

The role of school food has been highlighted during COVID‐19, and with this, there have been calls for FSM eligibility to be reviewed and expanded, not least by the National Food Strategy (Dimbleby, 2021). The continued need for improved and consistent monitoring of school food across the UK remains, as does the need to evaluate the impact of school food on children's diets.

## CONFLICT OF INTEREST

The authors have no conflict of interests to declare.

## AUTHOR CONTRIBUTIONS

The authors’ responsibilities were as follows: conceptualization, RM, SS and AA; manuscript draft, RM; manuscript revision, SS, AA, JW, SB, MN. All authors read and approved the final manuscript.

## Data Availability

Data sharing not applicable to this article as no datasets were generated or analysed during the current study
